# Genomic insights into *Solea solea* gut-borne *Enterococcus faecalis* for the development of new probiotics in aquaculture

**DOI:** 10.3389/fvets.2026.1778532

**Published:** 2026-04-22

**Authors:** Nadia Hussain, Fatima Muccee, Afreen Fatima Mirza, Naeem Mahmood Ashraf, Amal H. I. Al Haddad

**Affiliations:** 1 Department of Pharmaceutical Sciences, Al Ain Campus, Al Ain University, Abu Dhabi, United Arab Emirates; 2 Department of Pharmaceutical Sciences, College of Pharmacy, Al Ain University, Al Ain Campus, Al Ain, United Arab Emirates; 3 University of Punjab, School of Biochemistry and Biotechnology, Lahore, Pakistan; 4 Sheikh Shakhbout Medical City (SSMC), PureHealth, Chief Operations Office, Abu Dhabi, United Arab Emirates

**Keywords:** binning, comprehensive genome annotation, *Enterococcus faecium*, PATRIC, RASTk pipeline

## Abstract

Dietary manipulations using probiotics may contribute to the sustainable development of aquaculture. For probiotic applications, their traits, safety profiles, and functional characteristics should be explored. Whole genome sequencing (WGS) can be an informative tool in this regard. We initiated this study to obtain genomic insights into *S. solea* gut-associated bacteria. Bacteria (*n* = 20) exhibiting probiotic characteristics were subjected to DNA extraction. A mixture comprising equimolar concentrations of each bacterial DNA was prepared and analyzed through WGS. BV-BRC, metaSPAdes, MetaBAT2, DAS Tool, and PATRIC were used for taxonomic profiling, metagenome assembly, genome binning, comprehensive genome annotation, and subsystem analysis, respectively. For tree construction, MUSCLE and RaxML were employed. Fourteen bins comprising *Actinomycetota*, *Bacillota*, *Bacteroidota,* and *Pseudomonadota* were generated. Among these, the bin comprising the genome of *Enterococcus faecium* was selected. Its genome comprises 129 contigs with 2,944 coding sequences (CDSs). Genes associated with metabolism, protein processing, stress response, defense and virulence, cellular processes, and cell envelope were identified. Pathways identified included fatty acid and ketone body biosynthesis, glycerolipid and glycerophospholipid metabolism, linoleic acid metabolism, and self-defense mechanisms. This study confirmed the probiotic efficiencies of *E. faecium*. Hence, this bacterium might be employed as a fish feed supplement in aquaculture.

## 1 Introduction

One of the fastest-growing sectors of animal food production is aquaculture, which plays a pivotal role in international trade and helps address food security challenges. More than half of the fish consumed globally is produced by aquaculture (1). Its contribution is multifaceted at the national level as it provides high-quality protein, supports rural development, and opens up avenues for export earnings. In a study focusing on profitability analysis of recirculating systems for common carp, advanced farms were shown to generate substantial revenue, although they involve high capital costs that are difficult to balance in producer countries. Small-scale and even recreational fisheries further add indirect value through tourism and food security benefits. These studies provide evidence that aquaculture contributes significantly to local and national economies (2, 3). With its rapid expansion, concerns are emerging regarding the integration of sustainable development principles to ensure long-term viability and reduce environmental impact. Although aquaculture has a smaller ecological footprint than terrestrial protein sources, unsustainable practices can lead to significant risks, including habitat destruction, water pollution, and biodiversity loss (4). Analyses of aquaculture wastewater highlight the dangers of accumulating organic matter, nitrogenous compounds, and emerging contaminants that jeopardize surrounding ecosystems (5). Nutrient-rich effluents degrade local habitats through processes such as eutrophication. Intensive systems also consume large volumes of water and energy. These environmental pressures are further exacerbated by the rising cost of high-quality feed ingredients, which severely impacts productivity and profitability across diverse geographical settings (6).

In aquaculture, production efficiency is connected to economic feasibility, with special precautions implemented to mitigate industry risks. In high-density fish stocks, disease outbreaks can lead to devastating economic losses, and so broad-spectrum antibiotics have often been used in managing disease outbreaks (7). However, routine and even prophylactic administration of these antibiotics has greatly added to the spread of antimicrobial-resistant bacteria in aquatic environments and downstream food chains (8). Several studies have documented multidrug-resistant strains in finfish farms globally (9), while residual antibiotics have been shown to persist in sediments and water. These pose ecotoxicological risks and severely undermine public health policies developed to curb resistance. Antibiotic dependence is now well-recognized as a critical bottleneck for sustainable aquaculture (10).

Probiotics, as live microbial supplements, represent a bio-based strategy to reduce infection pressure without the limitations of chemotherapeutics. Strains with quorum-quenching activity can interfere with pathogenic cell-to-cell signaling, thereby reducing virulence gene expression and biofilm development (11). Studies have shown that *Bacillus velezensis* FLU-1 and its antimicrobial peptide LCI significantly lower mortality in fish challenged with *Aeromonas hydrophila*, highlighting the practical potential of probiotic interventions (12). Using such functional feeds also aligns with consumer demand for residue-free seafood while supporting immune modulation and growth performance (13).

A bacterium, to qualify as a probiotic, must exhibit certain functional, technological, and safety characteristics for its application in aquaculture (14). These bacteria should be compatible with the host, non-pathogenic, not resistant to antibiotics, and non-producers of toxic metabolites like biogenic amines. They should be capable of withstanding the GIT conditions, such as acidity and bile salts, demonstrate epithelial cell adhesion and colonization, antimicrobial activity, immunomodulation, and high stability and viability during processing (15). In addition, these bacteria must exert a positive impact on feed conversion ratios (FCR), specific growth rate (SGR), and body weight of host fishes through the production of exoenzymes, which improve nutrient digestibility and feed utilization (16). Such bacteria efficiently exclude pathogenic species from the gut through cell surface hydrophobicity and auto-aggregation. Aquaculture-related probiotics must also be capable of improving water quality through bioremediation of plankton remnants and fish excreta, as well as reduction of ammonia and nitrite (17).

This study has targeted the probiotics of *Solea solea* fish gut, which is commonly known as sole fish. It is a bentophagous fish with a sedentary lifestyle and exhibits batch spawning. At the stage of metamorphosis, it switches its behavior from diurnal to nocturnal (18). *S. solea* has a European origin. However, its demand has been increasing in Asian aquaculture due to its contribution to the expansion of their marine fish portfolio. Due to aquaculture’s suitability, adaptability, and high market demand in Pakistan, sole farming has become a major avenue of income generation. Its aquaculture has been facing challenges, including high mortality rates of larval stages and susceptibility to pathogens, making it a suitable host for probiotic studies (19). Probiotic administration in sole aquaculture not only improves digestive efficiency and growth rate but also enhances intestinal villi height, integrity of epithelium, reduces heat shock protein expression, thereby lowering stress levels in larvae, and boosts immunity, leading to higher survival rates at larval stages (17).

Whole-genome sequencing (WGS) has become vital for selecting and characterizing fish probiotics (20). Comparative WGS analyses pinpoint genes for adhesion, biofilm formation, vitamin synthesis, and bacteriocin production (21). This greatly enables the precise prediction of probiotic functionality and safety. Additionally, *de novo* fish genome assemblies are accelerating the identification of host–microbe interaction loci. This can facilitate probiotic strain–host matching and the development of trait improvement programs in aquaculture. In one study, the complete genome of *Bacillus pacificus* CR121 clarified its metabolic versatility and host-protective traits and helped pave the way for targeted formulation in carp and tilapia farming (22). Recent strain-level analyses have given insight into specific probiotic candidates and their functional attributes in aquaculture species. A study focusing on *Lysinibacillus* and *Enterobacter* strains from triploid cyprinid fish showed genomes with genes for biofilm formation, hydrophobicity, aggregation, nutrient metabolism, and potential pathogen inhibition. These traits are vital for host colonization, immune interaction, and pathogen resistance (23).

Considering the importance of WGS in probiotics characteristics exploration, we initiated this study to obtain insights into the genome of the bacterium *Enterococcus faecalis* isolated from *the gut of S. solea*. We hypothesized that we might identify genes and pathways associated with probiotic characteristics in bacteria isolated from the gastrointestinal tract (GIT) of healthy *S. solea* fish through WGS.

## 2 Methodology

### 2.1 Isolation of fish gut-associated bacteria

For isolation of fish gut-associated bacteria, fish *S. solea* (sole fish) was purchased from a commercial fish supplier in Lahore located at Qadimi Shehar. A total of 10 healthy, asymptomatic fish lacking visible signs of illness and with intact mucous/skin and intact gills were selected. Fish weighing 170–230 g and measuring 25–30 cm in length were selected. To avoid tissue damage, dead fish were handled and transported following the Guidelines of CPCSEA for Experimentation on Fishes. The fish were dissected by veterinary experts. Following this, washing of the gastrointestinal tracts (GIT) was performed in sterilized distilled water and phosphate buffer saline (PBS). The intestinal posterior portion was homogenized in 100 mL of Ringer’s solution. The homogenate was kept under shaking conditions at 150 rpm and at 37 °C overnight. To obtain the bacterial isolates, serial dilutions up to sixfold were prepared from the Ringer’s solution. These dilutions were spread on Petri plates containing Mueller–Hinton Agar (MHA) medium. Plates were incubated at 37 °C and for 24 h to allow bacterial growth (23). The CFUs were calculated. After the colonies appeared, the streak plate method was employed to obtain the purified colony. Isolated colonies were stored in the form of 30% glycerol stocks at −20 °C.

### 2.2 Morphological, biochemical, and molecular characterization of the bacterium

The morphological characteristics, such as color, texture, shape, margins, and elevation, were observed. The bacterium was initially identified on the basis of 16S rRNA gene sequencing. For this purpose, DNA was extracted as mentioned in sub-section 2.4 and amplified via PCR using previously documented primers (24, 25). Amplicons were analyzed via Sanger sequencing at Macrogen, Republic of Korea. To biochemically test the isolate, catalase, HCN, cellulase, pectinase, and chitinase production tests; mannitol salt agar (MSA) test; and sucrose, glucose, and lactose fermentation assays were carried out (26–30). Growth curve analysis was performed by measuring the OD^600^ of synchronized bacterial cultures at different time intervals.

### 2.3 Probiotic characterization of bacterium

Probiotic characterization involved the bile salt, NaCl, pH, and simulated gastric fluid tolerance assays; intestinal cell adhesion test; hemolytic and cholesterol assimilation assays; and pathogens and antibiotics sensitivity tests. All experiments were run in triplicate.

#### 2.3.1 Bile salt tolerance assay

Bile salt tolerance potential of bacteria was assessed using autoclaved De Man-Rogosa-Sharpe (MRS) broth supplemented with bile salts (0.3 g) (Himedia, Cat # RM008-500G). The medium (5 mL) was inoculated with an overnight-grown bacterial culture (OD^600^ = 0.1). Afterward, the tubes were incubated (150 rpm and 37 °C) until the exponential phase was achieved. At 0 and 24 h, OD^600^ was measured (31).

#### 2.3.2 NaCl tolerance assay

MRS media with different NaCl (WEL GENE Precision Solution™, Cat # ML011-01) concentrations, namely 0.2, 2, and 5 g, were prepared separately and autoclaved. Media were inoculated with bacterial cultures with OD^600^ = 0.1. Afterward, OD^600^ was measured at 0 h. Test tubes were then kept in a shaking incubator (37 °C and 150 rpm) for efficient growth. After 24 h, OD^600^ was measured again to check the tolerance potential of NaCl in the isolate (31).

#### 2.3.3 pH tolerance assay

1 N NaOH and 1 N NaCl were used to prepare the MRS medium of three different values of pH, that is, 2, 3, and 5. It was followed by inoculation with freshly grown bacterial culture and measurement of OD^600^ at 0 h. Test tubes were then placed in a shaking incubator (37 °C and 150 rpm) for efficient growth. After 24 h, OD^600^ was measured again to assess the growth of isolates in this study at different pH levels. The experiment was performed in triplicate for accurate results. For comparison of the growth potential of bacteria with each other and with the control, a bar graph was plotted (31).

#### 2.3.4 Simulated gastric fluid tolerance assay

Synthetic gastric fluid was prepared by dissolving 0.0061 g KH_2_PO_4_, 0.0256 g KCl, 0.105 g NaHCO_3_, 0.1375 g NaCl, 0.0012 g MgCl_2_ (H_2_O)_6_, 0.0024 g (NH_4_)_2_CO_3_, 0.0284 g HCl, and 0.0011 g CaCl_2_(H_2_O)_2_ in distilled water (50 mL). Fluid was passed through a porous membrane of 0.2 μm pore size. To the gastric fluid (15 mL), 0.01 mL of 0.3 M CaCl_2_, 3.2 mL of pepsin solution, and 1.8 mL of distilled water were added. The pH of the medium was set at 2. Prepared synthetic gastric medium was supplemented with the isolates of this study in the ratio 1:10 and kept at room temperature for 3 h followed by streaking on MHA agar plates. Plates were incubated at 37 °C overnight. Isolated bacterial strain growth was observed in the form of discrete colonies. Results were compared with the negative control (32).

#### 2.3.5 Cell adhesion assay

Approximately 1 cm of the posterior part of the fish was taken and placed in an overnight bacterial cell suspension for 30, 60, and 90 min. After a specified time of incubation, the intestinal pieces were placed on freshly prepared autoclaved MHA agar plates after maceration and then placed in an incubator at 37 °C for 24 h. Growth was observed to enumerate the CFUs and estimate the adhesion potential of the bacteria (33).

#### 2.3.6 Hemolytic assay

Fresh overnight cultures of the isolates used in this study were streaked on blood agar medium in Petri plates, followed by incubation at 37 °C until the exponential phase was achieved. Afterward, the hemolytic activity and its type (*α*, *β*, or *ϒ*) exhibited by the isolates were assessed by analyzing the color change (34).

#### 2.3.7 Cholesterol assimilation assay

The cholesterol breakdown potential of the isolate in this study was assessed using the cholesterol liquicolor kit (Pathozymes Diagnostics, India) via the CHOD-PAP method. A total of 1 mL of probiotic culture and 10 μL of cholesterol standard were added to the cuvette, mixed, and allowed to stand at room temperature for 5 min according to the manufacturer’s instructions. A total of 1 mL of cholesterol reagent and 10 μL cholesterol standard were added to the cuvette, taken as the standard. OD of the test sample and the standard was measured at 505 nm against a standard cuvette (35). Cholesterol assimilation level was evaluated by using the following formula:

$$\text{Cholesterol concentration}\;(\mathrm{mg}/\mathrm{dl})=\frac{\begin{array}{l} \text{Absorbance value of}\;\\ \text{test sample} \end{array}}{\begin{array}{l} \text{Absorbance value of}\;\\ \text{standard} \end{array}}\times 200$$

#### 2.3.8 Pathogens resistance assay

The pathogen resistance of the bacterium in this study was assessed using *Pseudomonas aeruginosa* and *Staphylococcus aureus*. Overnight cultures of isolates were added to freshly prepared MHA media contained in a Petri plate using the spread plate method. Wells were formed in the medium using a cork borer. Freshly grown pathogen culture was added to agar wells, and this was followed by incubation performed at 37 °C for 24 h (36). The extent of sensitivity of bacteria was assessed by measurement of the diameter of zones of inhibition.

#### 2.3.9 Antibiotic sensitivity assay

Different antibiotics were used to evaluate the antibiotic sensitivity profile of the isolates of this study: 250 mg of erythromycin, ampicillin, and azithromycin, and 500 mg of ciprofloxacin and amoxil. Antibiotic disks were prepared manually. The disk diffusion method was used to perform this test. The effect of antibiotics on the probiotic isolate was evaluated by measuring the zones of inhibition. Different dilutions of antibiotic solutions (10, 20, and 30 μL) were used to evaluate the extent of bacterial sensitivity (31).

### 2.4 Whole genome sequencing

#### 2.4.1 Sample preparation and quality control analysis of DNA

Initially, we isolated and analyzed 20 bacteria for probiotic characterization. The DNA of these bacteria was extracted using the organic method of DNA extraction. This method employed the use of phenol:chloroform:isoamylalcohol (37). Quality of DNA was confirmed via agarose gel electrophoresis. Equimolar DNA (100 ng) of each of these 20 bacteria was mixed to produce a mixture. This sample, comprising a DNA mixture, was sent to Macrogen, Inc., Republic of Korea, for WGS analysis.

#### 2.4.2 Library construction and Illumina sequencing

The sequencing libraries were prepared according to the manufacturer’s instructions of TruSeq Nano DNA High Throughput Library Prep Kit (Illumina, Inc). Briefly, 100 ng of genomic DNA was sheared using adaptive focused acoustic technology (Covaris), and the fragmented DNA was end-repaired to create 5′-phosphorylated, blunt-ended dsDNA molecules. Following end-repair, DNA was selected based on size with a bead-based method. These DNA fragments go through the addition of a single “A” base and ligation of the TruSeq DNA UD Indexing adapters. The products were then purified and enriched with PCR to create the final DNA library. The libraries were quantified using qPCR according to the qPCR Quantification Protocol Guide (KAPA Library Quantification kits for Illumina Sequencing platforms) and qualified using the Agilent Technologies 4,200 TapeStationD1000 screentape (Agilent Technologies). Then, paired-end (2 × 150 bp) sequencing was performed by Macrogen using the NovaSeq (Illumina).

### 2.5 Submission and bioinformatics analysis of FastQ files

The FastQ files obtained after WGS were submitted to NCBI Sequence Read Archives (SRA) to get the Bioproject and Biosample Accession IDs assigned.

### 2.6 FastQC tool

To generate the quality report of raw FastQ files, the FastQC tool was used, and the files were run from the command line via fastqc your_file.fastq.gz. This analysis provided an HTML and a zip file for each given input file. The key parameters evaluated included per-base sequence quality, per-sequence GC content, adapter content, sequence duplication levels, per-base N and sequence content, sequence length distribution, over-represented sequences, and basic statistics, such as total number of reads, their length, and GC content percentage.

### 2.7 Trimmomatic tool

For preprocessing of NGS paired-end reads, Trimmomatic, a cross-platform tool licensed under GPL V3^1^ was used. This platform is used to remove the technical sequences, such as primers and adaptors, from raw sequencing reads. Key trimmomatic parameters considered were seed mismatches (02), simple clip threshold (10), Trailing, Leading, Sliding Window, Headcrop, and Minlen. Operational parameters included threads, quality score (phred64), and paired-end (PE) data.

### 2.8 BV-BRC taxonomy tools

Bacterial and Viral Bioinformatics Resource Center (BV-BRC)^2^ is a taxonomic classification web-based, command-line service, which performs identification of the taxonomy of the sample using the Kraken2 tool and NCBI taxonomy. Kraken2 assigns reads to taxonomic bins. Key parameters considered during taxonomy analysis were sequencing data input, database selection, identification of species, platform of sequencing, size of insert, read orientation, output control, and taxonomic resolution.

### 2.9 MetaSPAdes

MetaSPAdes produces high-quality *de novo* assemblies from metagenomics datasets. It initially constructs a de Bruijn graph from sequence reads using different k-mer sizes. It differentiates inter-species repeats and sequences with different coverage depths. To predict the long genomic fragments in the metagenome, it constructs assembly graphs. Input parameters taken into consideration during metagenome assembly were paired-end reads, assembly directory, K-mer sizes, thread usage, and memory limit.

### 2.10 MetaBAT2 and DAS tools

For reconstructing microbial genomes from complex metagenomics data, two bioinformatic software tools, Metagenome Binning based on Abundance and Tetranucleotide frequency (MetaBAT2) and Dereplication, Aggregation and Scoring (DAS) tools were employed. MetaBAT2 is a *de novo* binning software that clusters contigs into genomes. DAS tool refines the results produced by MetaBAT2 and provides a non-contaminated genome set, known as metagenome-assembled genomes (MAGs) of high quality. Key parameters considered while using MetaBAT2 were minimum contig size for binning (−-minContig), good contigs percentage (−-maxP), minimum edge score (−-minS), maximum number of edges per node (−-maxEdges), TNF probability cutoff (−-pTNF), minimum mean contig coverage in each library (−-minCV), and minimum total effective mean coverage (−-minCVSum). Key considerations of parameters in the case of the DAS tool were input bins, contig FastA, score threshold, and algorithm integration.

### 2.11 CheckM

CheckM analyzes the quality of genome assemblies of microbes through comparison of the copy number of single-copy marker genes specific to genomes lineage to the reference genome taxonomic group. To indicate the contamination from another microbe, it identifies marker genes that are duplicated or missing. Key metrics considered were completeness estimate (marker gene presence, collocated marker sets, and marker set size), redundancy estimate (multicopy marker genes, contamination percentage, and strain heterogeneity), lineage-specific marker gene selection (phylogenetic placement, lineage-specific sets, and parent node selection), and input data requirements (genome assembly in FASTA format).

### 2.12 Comprehensive genome analysis meta-service, PATRIC

For genome annotation, the assembled genomes of *E. faecium* bins were submitted to a comprehensive genome analysis (CGA) meta-service and PATRIC (38). QUAST quality analysis parameters, such as the number of contigs, GC content, contig L50, genome length, and number of contigs with N50 were predicted. Parameters taken into consideration in PATRIC meta service included genome assembly and QC (reads processing, assembly and QC metrics like N50, genome length, contig count, and GC content).

For annotation of the genome, the Bacterial and Viral Bioinformatics Resource Center (BV-BRC) RASTk pipeline was consulted (39). BV-BRC uses Rapid Annotation using Subsystem Technology (RAST) tool kit (RASTtk) to predict features of a genome, such as protein coding sequences (CDSs), repeat regions, tRNA, partial CDS, and rRNA. Features of annotated proteins included the number of hypothetical proteins, proteins functional and non-functional, EC number, GO, pathway, PATRIC genus-specific family (PLfam), and PATRIC cross-genus family (PGfam) assignments.

### 2.13 Phylogenetic analysis

The National Center for Biotechnology Information (NCBI) staff manually select and categorize reference and representative genomes, which they consider to be of high quality and importance to the research community. PATRIC provides the reference and representative genomes and includes them in the phylogenetic analysis that is part of the Comprehensive Genome Analysis report. The closest reference and representative genomes were identified by Mash/MinHash (40). PATRIC global protein families (PGFams) (41) were selected from these genomes to determine the phylogenetic placement of this genome. The protein sequences from these families were aligned with MUSCLE (42), and the nucleotides for each of those sequences were mapped to the protein alignment. The joint set of amino acid and nucleotide alignments was concatenated into a data matrix, and RaxML (43) was used to analyze this matrix, with fast bootstrapping used to generate the support values in the tree (44).

### 2.14 Specialized genome annotation

The Genome Annotation Service in PATRIC uses the k-mer-based AMR gene detection method, which utilizes PATRIC’s curated collection of representative AMR gene sequence variants and assigns to each AMR gene functional annotation, broad mechanism of antibiotic resistance, drug class, and, in some cases, specific antibiotic it confers resistance to. A point of consideration here is that the presence of AMR-related genes (even full-length) in a given genome does not directly imply an antibiotic-resistant phenotype. It is important to consider specific AMR mechanisms, especially the absence/presence of SNP mutations conveying resistance.

PATRIC annotation was also employed to analyze the subsystems unique to the genome of *E. faecius*. A subsystem is a set of proteins that together implement a specific biological process or structural complex (45).

## 3 Results

### 3.1 Isolation and probiotic screening of *E. faecium*

Isolation of *E. faecium* from *S. solea* gut through the serial dilution method revealed that the number of CFUs was 40 × 10^−5^. Morphological analysis demonstrated off-white colonies with creamy texture and irregular shape. Margins and elevations of colonies were lobate and raised, respectively. The sequence obtained through molecular characterization of the bacterium based on the 16S rRNA gene was submitted to the NCBI database under the Accession ID PQ002492. Its strain was designated as SBBPro7, where SBB stands for School of Biochemistry and Biotechnology, and where isolation and experimental characterization were performed. Pro stands for probiotics, and 7 is the number of discovery. Biochemical characterization revealed positive results for catalase and MSA tests; HCN, cellulose, and pectinase production tests; and glucose, sucrose, and lactose fermentation assays, but negative for the chitinase test. The bacterium was fast growing with a log phase starting after 24 h and lasting until 27 h.

Assessment of probiotic features showed the bacterial growth potential in the presence of bile salts, with OD^600^ 1.91 ± 0.02 at log phase, and good tolerance against NaCl with OD^600^ of 1.15 ± 0.04 at log phase. At pH = 5, average growth was demonstrated by *E. faecium* with OD^600^ 0.85 ± 0.04 at log phase. The bacterium exhibited considerable growth in the presence of simulated gastric medium. The intestinal cell adhesion efficiency, observed at 30, 60, and 90 min was 40 ± 0.577, 57 ± 1.154, and 118 ± 1.154, respectively. The bacterium demonstrated gamma hemolysis. Isolates assimilated 83.44 mg/dL of cholesterol during the cholesterol assimilation assay. The isolate was resistant to *S. aureus* and *P. aeruginosa* and sensitive to five different antibiotics, such as amoxil, erythromycin, velosef, azithromycin, and ciprofloxacin (Table 1). All these characteristics confirmed the probiotic potential of *E. faecium* SBBPro7.

**Table 1:** Morphological, biochemical, and probiotic characterization of *E. faecium* isolated from *S. solea* fish gut.

No.	Assays performed for characterization	Characteristics observed	
1	Number of CFUs	40 × 10^−5^	
2	Morphological characteristics	Off-white color with creamy texture, irregular shape, lobate margins, and raised elevation	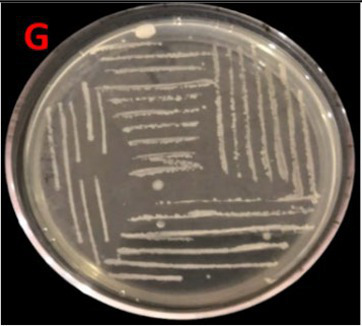
Biochemical characteristics			
3	Catalase production test	+ve	
4	Mannitol salt agar test	+ve	
5	HCN production test	+ve	
7	Glucose fermentation test	+ve	
8	Sucrose fermentation test	+ve	
9	Lactose fermentation test	+ve	
10	Cellulase production test	+ve	
11	Pectinase production test	+ve	
12	Chitinase production test	-ve	
13	Growth curve analysis	Lag phase (0–24 h), Log phase (24 < till 27 h), S phase (27 < till 48 h), D phase (48 <)	
Probiotics characteristics analysis			
14	Bile salts tolerance assay	OD^600^ 1.91 ± 0.02 at log phase	
15	NaCl tolerance assay	OD^600^ 1.15 ± 0.04 at log phase	
16	pH tolerance assay	OD^600^ 0.85 ± 0.04 at log phase	
17	Gastric fluid tolerance assay	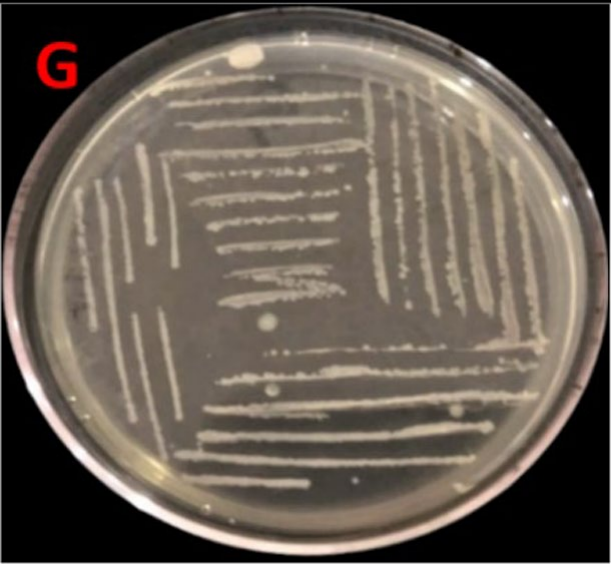	
18	Cell adhesion assay	At 30 min = 40 ± 0.577 At 60 min = 57 ± 1.154 At 90 min = 118 ± 1.154	
19	Hemolytic test	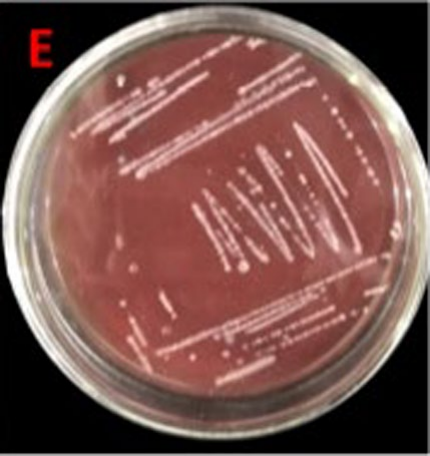	
20	Cholesterol assimilation test	83.44 mg/dL	
21	Pathogens resistance assay	Resistant to *S. aureus* and *P. aeruginosa*	
22	Antibiotic sensitivity test	Sensitive to amoxil, erythromycin, velosef, azithromycin, and ciprofloxacin	

### 3.2 WGs-based genomic interpretation

#### 3.2.1 Taxonomic characterization of the sample and selection of *E. faecium*

Taxonomic characterization helped us to identify four phyla in the DNA sample, including *Actinomycetota*, *Bacillota*, *Bacteroidota,* and *Pseudomonadota*. Fourteen bins were generated for these four phyla (Supplementary Figure 1). Bacteria identified in the sample were *Enterococcus faecium*, *Lactococcus formosensis*, *L. garvieae*, *Citrobacter werkmanii*, *Enterobacter cloacae*, *Escherichia coli*, *Klebsiella aerogenes*, *K. pneumoniae*, *Proteus terrae,* and *Proteus vulgaris*.

Among these, the bin representing the genome of *E. faecium* was selected for this study. The majority of the bacteria identified in the sample, such as *Proteus*, *Klebsiella,* and *Escherichia,* are pathogenic. In addition, in comparison with the rest of the bacteria, *E. faecium* has superior digestive survival due to high viability in bile salts and acid-rich environments and increased capacity of epithelial cells adhesion, biofilm formation, and outcompeting the pathogens (46). Being the most frequently isolated bacterium from the guts of healthy fish, it exhibits autochthonous potential (47). Due to the production of enterocin P, this bacterium demonstrated antipathogenic activity against aquaculture pathogens, such as *Vibrio*, *Steptococcus agalactiae*, *E. coli,* and *Aeromonas hydrophila* (48). Considering all these positive facts, *E. faecium* seems to offer a broader range of benefits in aquaculture settings.

Bin generated for this bacterium met the quality criteria of fine consistency (> = 87%) and completeness (> = 80%), that is, 97.3 and 100% for *E. faecium*. The values of coarse consistency, fine consistency, contig count, DNA size, and mean coverage identified in this bin were 99.8, 97.3, 100%, 296, 3,011,104 bp, and 260.18, respectively.

#### 3.2.2 QUAST quality analysis and genome annotation

In *E. faecium*, the genome assembly showed the following features: contigs (129), GC content (38.00%), contig L50 (14), genome length (2,836,039 bp), and contig N50 (75082) (Supplementary Table S1).

#### 3.2.3 Genome annotation

The *E. faecium* bin 12 genome was annotated using the RAST tool kit (RASTtk) and assigned a unique genome identifier of 1351.12018. This genome is in the superkingdom bacteria and was annotated using genetic code 11. The taxonomy of this genome was found as cellular organisms > Bacteria > *Bacillati* > *Bacillota* > *Bacilli* > *Lactobacillales* > *Enterococcaceae* > *Enterococcus* > *Enterococcus faecalis*. Genome annotation identified 2,944 CDS, 33 tRNA, and 0 rRNA. The annotated features are summarized in Supplementary Table S2.

The annotation included 887 hypothetical proteins and 2057 proteins with functional assignments (Supplementary Table S3). The proteins with functional assignments included 684 proteins with Enzyme Commission (EC) numbers, 560 with Gene Ontology (GO) assignments (49), and 462 proteins that were mapped to KEGG pathways (50). PATRIC annotation includes two types of protein families (38), and this genome has 2,674 proteins that belong to the genus-specific protein families (PLFams) and 2,813 proteins that belong to the cross-genus protein families (PGFams).

**Figure 1:**
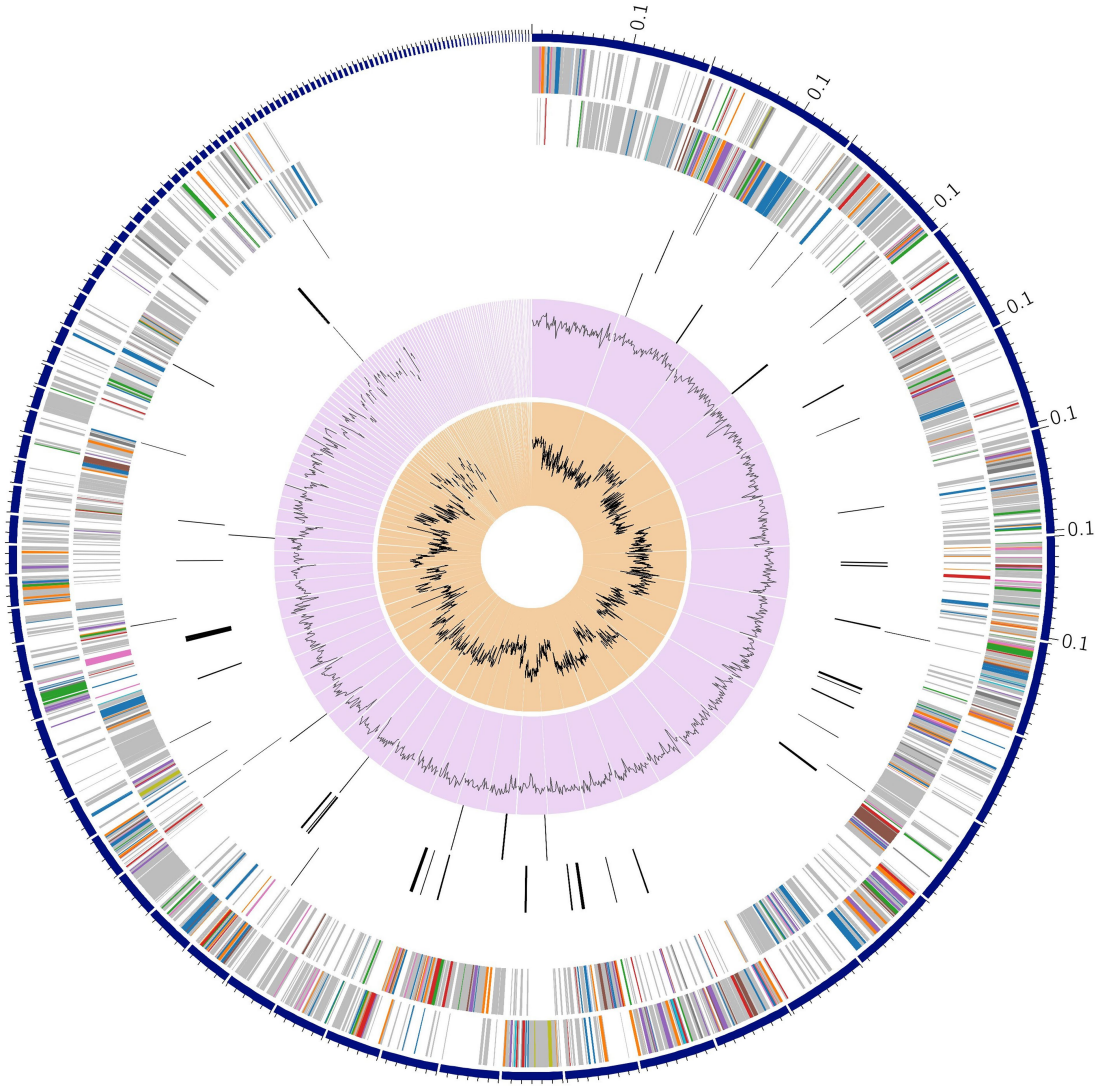
Circos plot for circular graphical display of genome annotations distribution in *E. faecium*.

The graphical distribution of the genome annotation of the bacterium is represented in Figure 1. In the Circos diagram, rings from outer to inwards represent the contigs, CDS on the forward and reverse strands, RNA genes, CDS with homology to known antimicrobial resistance genes and virulence factors, GC content, and GC skew. The colors of the CDS on the forward and reverse strands indicate the subsystem that these genes belong to (see Subsystems below).

#### 3.2.4 Specialized genome annotation

Many of the genes annotated showed homology to known transporters (51), virulence factors (52, 53), drug targets (54, 55), and antibiotic resistance genes (56). The number of genes and the specific source database where homology was found are provided in Table 2. Antibiotic resistance-associated genes were identified through databases CARD (11), NDARO (3), and PATRIC (36). Genes encoding drug targets were revealed via databases DrugBank (10) and TTD (2). Twenty-three genes were found associated with transporters through the TCDB database. Virulence factors encoding genes were identified through PATRIC_VF (1), VFDB (4), and Victors (9).

**Table 2:** Number of specialty genes identified in *E. faecium* and specific source databases where homology was found.

Specialty genes	Source database	Genes
Antibiotic resistance	CARD	11
Antibiotic resistance	NDARO	3
Antibiotic resistance	PATRIC	36
Drug target	DrugBank	10
Drug target	TTD	2
Transporter	TCDB	23
Virulence factor	PATRIC_VF	1
Virulence factor	VFDB	4
Virulence factor	Victors	9

AMR genes associated with AMR mechanisms were also annotated in this study. Only one antibiotic inactivation enzyme-encoding gene was identified, namely aminoglycoside acetyl transferase (AAC). Seventeen antibiotic target genes were found, such as alanine racemase (Alr), D-alanine-D-alanine ligase (Ddl), elongation factor-G (EF-G), elongation factor thermos unstable (EF-Tu), dihydrofolate reductase encoding gene (folA), dihydrofolate reductase associated with trimethoprim resistance (Dfr), DNA gyrase subunit A (gyrA), DNA gyrase subunit B (gyrB), NADH-dependent enoyl-ACP reductase (inhA), enoyl-(acyl-carrier-protein) reductase (fabl), isoacceptor tRNA (iso-tRNA), beta-ketoacyl ACP synthase-1 (kasA), UDP-N-acetylglucosamine enolpyruvyl transferase (MurA), rho protein encoding gene (rho), beta-subunit of DNA dependent RNA polymerase (rpoB and rpoC), and ribosomal protein S12 encoding gene (S12p) (Table 3).

**Table 3:** Antibiotic resistance genes (ARGs) annotated in the genome of *E. faecium* and corresponding ARG mechanism through PATRIC Genome Annotation Service.

Antibiotic resistance gene (ARG) mechanism	Genes
Antibiotic inactivation enzyme	ACC(6′)-la (and related AACs)
Antibiotic target in susceptible species	Alr, Ddl, EF-G, EF-Tu, folA, Dfr, gyrA, gyrB, inhA, fabl, iso-tRNA, kasA, MurA, rho, rpoB, rpoC, S12p
Antibiotic target-modifying enzyme	RlmA(II)
Antibiotic target protection protein	Lsa(A), Msr(C)
Gene conferring resistance via absence	gidB
Protein-altering cell wall charge conferring antibiotic resistance	GdpD, MprF, PgsA
Regulator modulating expression of antibiotic resistance genes	LiaF, LiaR, LiaS

#### 3.2.5 Phylogenetic analysis

**Figure 2:**
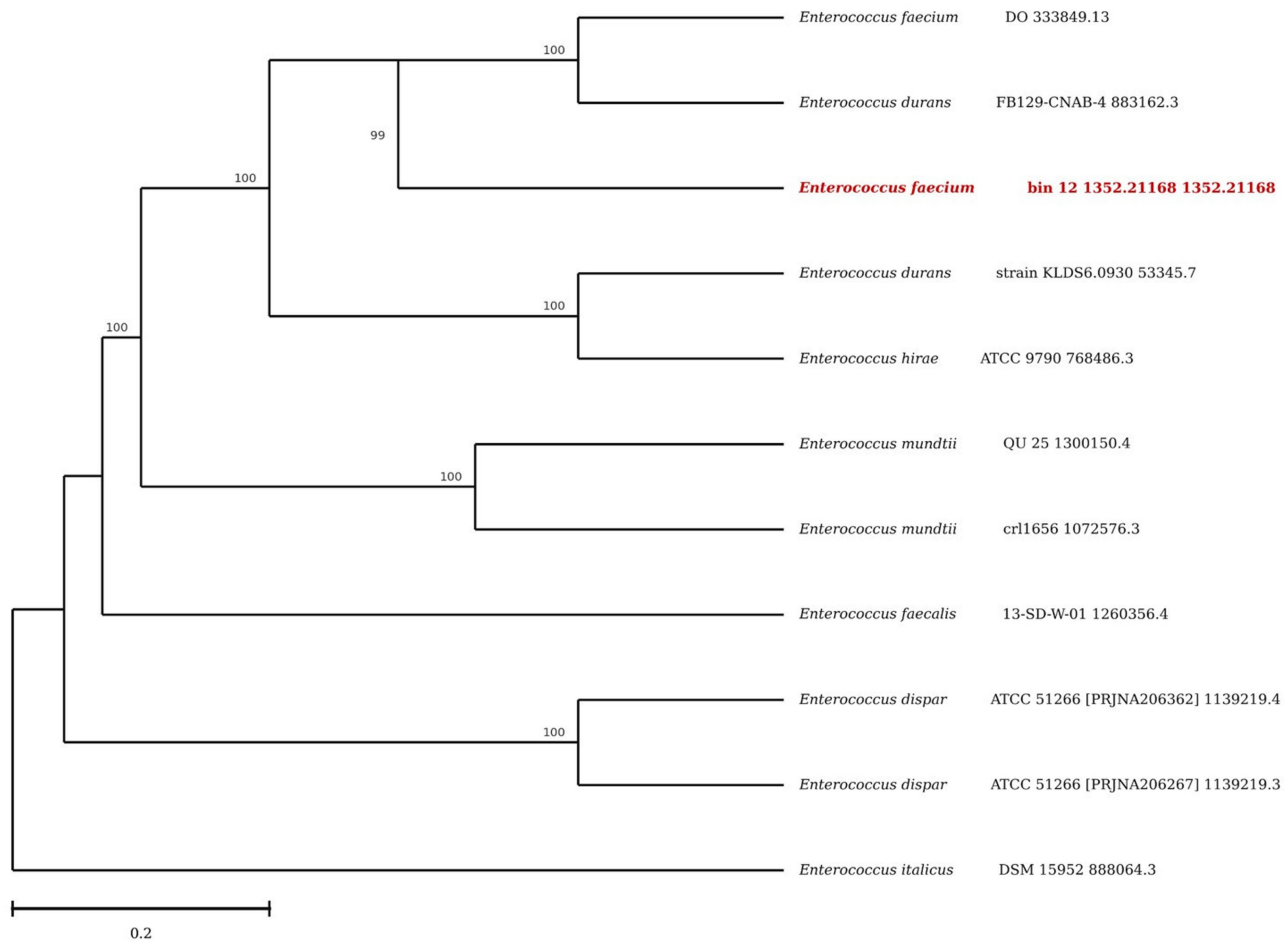
Phylogenetic analysis of *E. faecium* documented in this study.

*E. faecium* characterized in this study was closely related to *E. faecium* DO and *E. durans* FB129-CNAB-4, as they shared the same clade with a bootstrap value of 100. Bacterium was comparatively less related to *E. durans* strain KLDS6.0930, *E. hirae*, *E. mundtii* strains, and *E. faecium* 13-SD-W-01. Bacterium was distantly related to *E. dispar* and *E. italicus,* as these originated from distant branch points (Figure 2).

#### 3.2.6 Functional characterization- subsystem analysis

**Figure 3:**
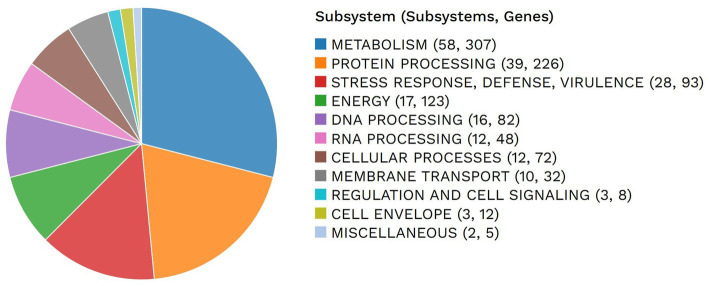
Subsystems of the *E. faecium* genome and number of associated genes identified in this study.

Subsystems identified in *E. faecium* included metabolism, protein processing, stress response, defense and virulence, energy, DNA processing, RNA processing, cellular processes, membrane transport, regulation, and cell envelope. The number of genes identified associated with these subsystems was 307, 226, 93, 123, 82, 48, 72, 32, 8, and 12, respectively. An overview of the subsystems for this genome is provided in Figure 3.

#### 3.2.7 Pathways and genes identified

Pathways identified in *E. faecium* included fatty acids, such as octanoic acid, decanoic acid, dodecanoic acid, tetradecanoic acid, hexadecanoic acid, octadecanoic acid and omega hydroxyl fatty acids synthesis, ketone bodies synthesis and degradation, steroid hormones synthesis, glycerolipid, glycerophospholipid and linoleic acid metabolism, and the streptomycin biosynthesis pathway. Genes identified in the bacterium involved in these pathways are shown in Table 4.

**Table 4:** Pathways and genes identified in this study *S. solea* gut-borne bacterium *E. faecium.*

EC #	Genes identified	Function
Fatty acids biosynthesis		
EC:2.3.1.179	Beta-ketoacyl-(acyl-carrier-protein) synthase II	Transfers acyl groups in fatty acid synthesis
EC:1.1.1.100	3-oxoacyl-(acyl-carrier-protein) reductase	Catalyzes reduction of the 3-oxoacyl group to a 3-hydrocyacyl group
EC:4.2.1.59	3-hydroxyacyl-(acyl-carrier-protein) dehydratase	Dehydration step of the dissociated (type II) fatty-acid biosynthesis system
EC:1.3.1.9	Enoyl-(acyl-carrier-protein) reductase (NADH)	Catalyzes an essential step in fatty acid biosynthesis, the reduction of the 2,3-double bond in enoyl-acyl-(acyl-carrier-protein) derivatives of the elongating fatty acid moiety.
Omega-oxo fatty acids synthesis		
EC:1.1.1.1	Alcohol dehydrogenase	Acts on primary or secondary alcohols or hemiacetals with very broad specificity; however, the enzyme oxidizes methanol much more poorly than ethanol.
Glycerolipid metabolism		
EC:2.3.3.10	Hydroxymethylglutaryl-CoA synthase	Catalyzes the conversion of acetoacetyl-CoA to hydroxymethylglutaryl-CoA
EC:2.7.1.31	Glycerate 3-kinase	Produce glycerate 2-phosphate (2PGA)
EC:4.2.1.28	Propanediol dehydratase	Converts 1,2-propanediol into propionaldehyde by catalyzing the removal of a hydroxyl group from the substrate.
EC:1.1.1.6	Glycerol dehydrogenase	Catalyzes the NAD-dependent oxidation of glycerol to dihydroxyacetone (DHA)
EC:2.7.1.30	Glycerol kinase	Catalyzes the conversion of glycerol to glycerol-3-phosphate (G3P), which is a key step for glycerol to enter the metabolic pathways of glycolysis, gluconeogenesis, and fat synthesis.
EC:3.1.1.23	Acylglycerol lipase	Hydrolyses glycerol monoesters of long-chain fatty acids
EC:2.7.1.107	Diacylglycerol kinase	Uses ATP to convert diacylglycerol (DAG) into phosphatidic acid (PA), acting as a central switch that regulates different bioactive lipid signaling pathways and contributes to lipid biosynthesis.
EC:2.4.1.-	Glycogen phosphorylase	Catalyzes the breakdown of stored glycogen into glucose-1-phosphate, providing a readily available carbon and energy source to sustain the cell during starvation or other stressful conditions
Glycerophospholipid metabolism		
EC:1.1.1.94	Glycerol 3-phosphate dehydrogenase	Facilitates the conversion of glycerol-3-phosphate to dihydroxyacetone phosphate while reducing ubiquinone.
EC:1.1.3.21	Glycerol-3-phosphate oxidase	Oxidizes glycerol 3-phosphate to glycerone phosphate and ‑, using dioxygen as an electron acceptor.
EC:1.1.5.3	Glycerol-3-phosphate dehydrogenase	Primarily synthesizes glycerol-3-phosphate, a precursor for phospholipid and glycerolipid biosynthesis, and plays a critical role in linking glycolysis to lipid metabolism.
EC:2.7.7.39	Glycerol-3-phosphate cytidylyltransferase	Catalyzes the transfer of a cytidyl group from cytidine 5′-triphosphate (CTP) to glycerol 3-phosphate (G3P) to form CDP-glycerol.
EC:2.7.7.41	Phosphatidate cytidylyltransferase, also known as CDP-diacylglycerol synthase	Catalyzes the reaction where cytidine triphosphate (CTP) and phosphatidic acid are converted into diphosphate and cytidine diphosphate-diacylglycerol (CDP-diacylglycerol)
EC:2.7.8.5	Glycerophosphate phosphatidyltransferase	Catalyzes the transfer of a phosphatidyl group from CDP-diacylglycerol to glycerol-3-phosphate, a key step in the biosynthesis of acidic phospholipids like phosphatidylglycerol and phosphatidylglycerol phosphate.
EC:2.7.8.-	Transferases	Catalyze the transfer of other substituted phosphate groups
EC:2.7.1.107	Diacylglycerol kinase (ATP)	uses ATP to convert diacylglycerol (DAG) into phosphatidic acid (PA), acting as a molecular switch by terminating DAG’s signaling while initiating PA’s signaling
EC:4.1.1.65	Phosphatidylserine decarboxylase	Converts phosphatidylserine (PtdSer) into phosphatidylethanolamine (PtdEtn) by removing a carboxyl group
EC:3.1.1.5	Lysophospholipase	Break down lysophospholipids, which are toxic to cell membranes, by hydrolyzing their ester bonds to produce fatty acids and water-soluble compounds like glycerophosphorylcholine (GPC).
EC:3.1.4.6	Deoxyribonucleate 3′-oligonucleotidohydrolase or deoxyribonuclease II	Hydrolyzes DNA at the 3″-phosphate bond.
EC:2.7.1.32	Choline kinase	Catalyzes the phosphorylation of choline using ATP to form phosphocholine and ADP
Linoleic acid metabolism		
EC:1.1.1.-	Alcohol dehydrogenase	Acts on primary or secondary alcohols or hemiacetals with very broad specificity
Bacterial self-defense		
EC:2.7.10.2	Tyrosine kinase/BY-kinases	Bacterial polysaccharide synthesis

### 3.3 Submission OF FASTQ files to SRA-NCBI

The sequences of bacteria identified in this study have been submitted to the NCBI database under the accession numbers given as follows: *Enterococcus faecium* (PQ002492.1), *Lactococcus formosensis* (PZ145238), *Lactococcus garvieae* (PZ145246), *Citrobacter werkmanii* (PZ145203), *Enterobacter cloacae* (PZ145205), *Escherichia coli* (PZ145204), *Klebsiella aerogenes* (PZ145234), *Klebsiella pneumonia* (PZ145235), *Proteus terrae* (PZ145245), and *Proteus vulgaris* (PZ145248). The FASTQ files obtained via WGS are submitted to SRA-NCBI under the BioProject number PRJNA1137886, BioSample number SAMN42644148, and SRA number SRR29890710.

### 3.4 Ethical approval

Ethical approval was granted by the Punjab University Institutional Review Board.

## 4 Discussion

Literature documents the use of *E. faecium* as a probiotic feed supplement in fishes like *Carassius auratus gibelio*, *Litopenaeus vannamei*, *O. niloticus, Petrophyllum scalare,* and *Paralichthys olivaceus* (46, 57–61). The bacterium was found to be associated with promoting immunity, growth, intestinal morphometry, and functionality of digestive enzymes.

A study has documented the Illumina HiSeq 2,500 system-based sequencing of *E. faecium*, isolated from the mucous of *O. niloticus*. This study identified genes associated with the same subsystems as documented in this study of a bacterium (62). Another study explored multidrug antimicrobial resistant genes like erm(B), lsa(A), lsA(E), lnu(B), dfr(G), tet(M), tet(L), aph(3′)-IIIa, ant(6)-la, spw, and sat4 in *E. faecium*, *E. faecalis,* and *E. durans* through WGS analysis (63). Another study targeted the complete genome sequence of *E. faecium* isolated from the gut *of Litopenaeus vannamei* and identified genes associated with nutrients and bacteriocin production (64). Bacteriocin-production genes were not identified in this study. However, the absence of virulence factors, esp. and hyl, in *L. vannamei*-associated bacterium is in accordance with this study because we did not find virulence-associated genes. Similarly, another study also reported the absence of cyclA and asa1 virulence genes. Same study, contrary to our findings, documented tetracycline and erythromycin resistance genes in *E. faecium,* analyzed through detailed phenotypic and genotypic characterization (65).

**Figure 4:**
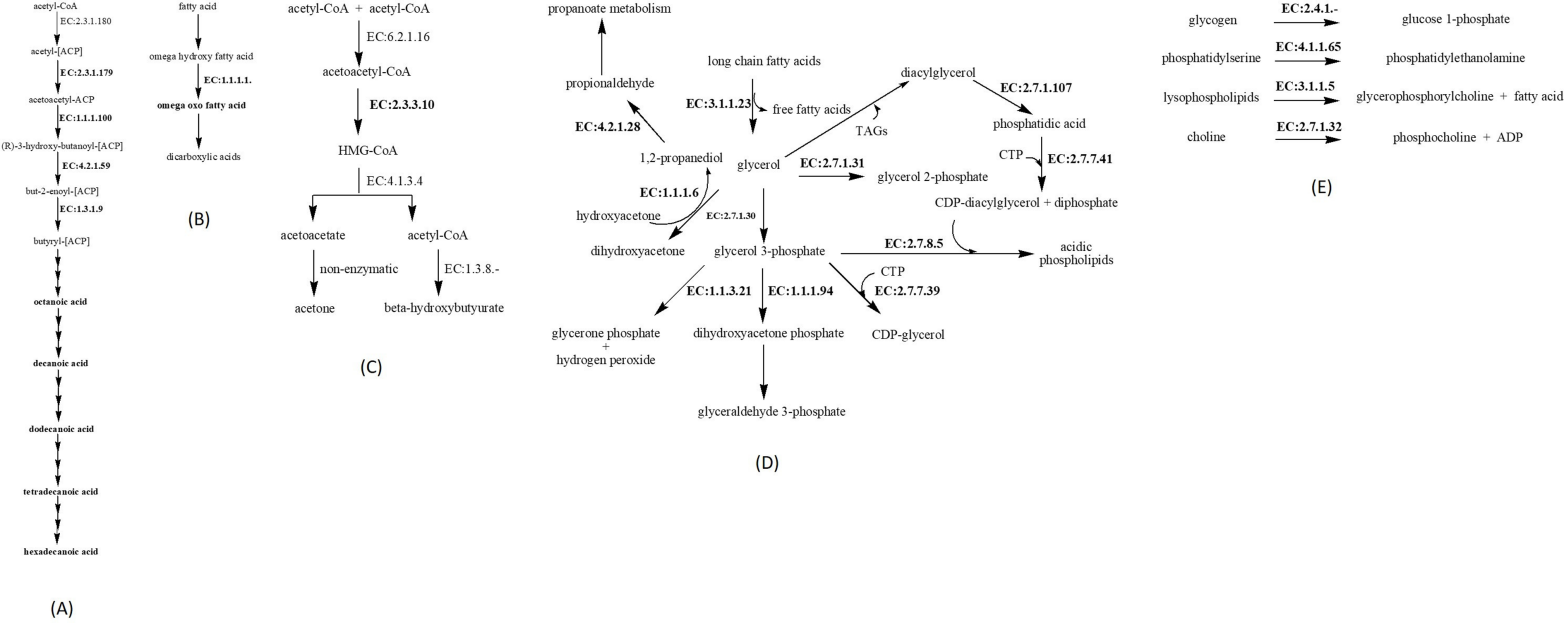
Enzymes identified in *E. faecium* via WGS and their associated pathways. **(A)** Enzymes associated with fatty acids synthesis, 2.3.1.179 = beta-ketoacyl-(acyl-carrier-protein) synthase II, 1.1.1.100 = 3-oxoacyl-[acyl-carrier-protein] reductase, 4.2.1.59 = 3-hydroxyacyl-(acyl-carrier-protein) dehydratase, 1.3.1.9 = enoyl-(acyl-carrier-protein) reductase (NADH). **(B)** Enzymes associated with synthesis of omega oxo fatty acid, 1.1.1.1 = alcohol dehydrogenase. **(C)** Enzymes associated with ketogenesis, EC:2.3.3.10 = hydroxymethylglutaryl-CoA synthase. **(D)** Enzymes associated with glycerolipid metabolism, EC:2.7.1.31: glycerate 3-kinase, EC:4.2.1.28: propanediol dehydratase, EC:1.1.1.6: glycerol dehydrogenase, EC:2.7.1.30: glycerol kinase, EC:3.1.1.23: acylglycerol lipase, EC:2.7.1.107: diacylglycerol kinase. Enzymes associated with glycerophospholipid metabolism, EC:1.1.1.94: glycerol 3-phosphate dehydrogenase, EC:1.1.3.21: glycerol-3-phosphate oxidase, EC:2.7.7.39: glycerol-3-phosphate cytidylyltransferase, EC:2.7.7.41: phosphatidate cytidylyltransferase, EC:2.7.8.5: glycerophosphate phosphatidyltransferase, EC:2.7.1.107: diacylglycerol kinase. **(E)** Enzymes associated with glycerophospholipid metabolism, EC:2.4.1.-: glycogen phosphorylase, EC:4.1.1.65: phosphatidylserine decarboxylase, EC:3.1.1.5: lysophospholipase, EC:2.7.1.32: choline kinase.

This study highlights the importance of *E. faecium* as a fish feed supplement through the identification of genes involved in fatty acid synthesis, glycerolipid, glycerophospholipid, and linoleic acid metabolism. Identification of genes associated with fatty acid synthesis in *E. faecium* is consistent with previous literature (66–68). In this study, we found the genes encoding the beta-ketoacyl-(acyl-carrier-protein) synthase II, 3-oxoacyl-(acyl-carrier-protein) reductase, 3-hydroxyacyl-(acyl-carrier-protein) dehydratase, enoyl-(acyl-carrier-protein) reductase (NADH), and alcohol dehydrogenase. Reactions catalyzed by these enzymes are shown in Figure 4. Fatty acid synthesis improves filet quality by increasing essential fatty acids, growth factors, and regulating lipid metabolism to prevent diseases (69).

The production of ketone bodies enables the availability of an alternate energy source under conditions of stress and starvation. Literature reports low ketone body levels in teleosts, grown under stress conditions, as compared to elasmobranchs, confirming fatty acids as their alternative energy source (70). The gene associated with ketogenesis identified in *E. faecium* encodes hydroxymethylglutaryl-CoA synthase, which catalyzes the condensation of acetyl-CoA and acetoacetyl-CoA to form HMG-CoA.

Finding the genes associated with glycerolipid and glycerophospholipid confirms the probiotic nature of *E. faecium*. The composition of these two lipid molecules serves as a physiological marker to confirm the survivability of *E. faecium* in harsh gastric conditions (71). It enables bacteria to confer health benefits to the host via the reduction of reactive oxygen species and hypocholesterolemic effects (72). Literature has documented an increase in the bioavailability of these two compounds in *Seriola lalandi* (yellowtail kingfish) and *S. rivoliana* (longfin yellowtail) under rearing conditions, contributed by their gut microbiota (73, 74). Genes identified associated with glycerolipid metabolism included glycerate 3-kinase, propanediol dehydratase, glycerol dehydrogenase, glycerol kinase, acylglycerol lipase, diacylglycerol kinase (ATP), and glycogen phosphorylase.

Genes associated with the metabolism of glycerophospholipid included glycerol 3-phosphate dehydrogenase, glycerol-3-phosphate oxidase, glycerol-3-phosphate dehydrogenase, glycerol-3-phosphate cytidylyltransferase, phosphatidate cytidylyltransferase, also known as CDP-diacylglycerol synthase, glycerophosphate phosphatidyltransferase, transferases, diacylglycerol kinase (ATP), phosphatidylserine decarboxylase, lysophospholipase, deoxyribonucleate 3′-oligonucleotidohydrolase or deoxyribonuclease II, and choline kinase.

Identification of linoleic acid production of *E. faecium* further strengthens its probiotic nature because this acid is used in the production of an anti-pathogenic, anti-carcinogenic, and anti-inflammatory functional lipid, conjugated linoleic acid (CLA) (75). CLA also plays an immunomodulatory and hypocholesterolemic role in the host (76).

Identification of BY-kinases in *E. faecium* indicates its strong potential to adapt to stressful environments. These tyrosine kinases are involved in the synthesis of polysaccharides, which serve in defense against environmental stress, desiccation, biofilm formation, and resistance to antimicrobials (77). Literature has documented these proteins in fish gut probiotics, such as species of *Bacillus* and *Lactobacillus* (78).

*E. faecium* is an opportunistic pathogen; therefore, its safety assessment is as critical as the probiotic efficacy itself. This study clearly demonstrated that this specific strain does not pose a biosecurity risk to the aquaculture environment or human consumers. The WGS did not identify the mobile genetic elements (MGEs), such as transposons and plasmids, ensuring the least potential for horizontal gene transfer of antibiotic resistance or virulence to human consumers or other microbes in the environment (79). Bacteria do not harbor the transferable acquired antibiotic resistance genes (80). Additionally, the strain characterized in this study is from an autochthonous source, that is, it has been isolated directly from the gut of healthy fish, which decreases the risks associated with non-native bacteria (81, 82). We also obtained the phenotypic evidence via *in-vitro* testing, that is, sensitivity to antibiotics, non-hemolytic nature, and survivability under gastric conditions, further strengthening the probiotic status of *E. faecium* (83). Hence, despite being recognized as an opportunistic pathogen, this study explored *E. faecium* and justified its use in aquaculture.

## 5 Conclusion

*E. faecium* documented in this study does not represent a public health concern because of the absence of plasmids, mutations, or acquired genetic elements in genes associated with antibiotic resistance. This results in a bacterium that exhibits phenotypic susceptibility to antibiotics. The absence of these resistance determinants makes *E. faecium* vulnerable to antibiotic treatment. However, its biosafety status must be assured via *in vivo* virulence testing in a suitable fish model in future studies. Moreover, due to the identification of genes associated with the synthesis of fatty acids, ketone bodies, linoleic acid, glycerolipids, and glycerophospholipids, the probiotic nature of the bacterium is evident. Therefore, *E. faecium* might be a good candidate for *in vivo* assessment of its impact on fish growth, body weight, and meat quality through in-feed administration.

## Data Availability

The datasets presented in this study can be found in online repositories. The names of the repository/repositories and accession number(s) can be found at: https://www.ncbi.nlm.nih.gov/, SRR29890710.

## Footnotes

1
http://www.usadellab.org/cms/?page=trimmomatic

2
https://www.bv-brc.org
