# Supplementary material for: Genomic insights into Solea solea gut-borne Enterococcus faecalis for the development of new probiotics in aquaculture
Source: Front Vet Sci. 2026 Apr 22;13:1778532. doi: 10.3389/fvets.2026.1778532 (PMC13143762; doi:10.3389/fvets.2026.1778532)
Supplement: Supplementary file 1 [file Data_Sheet_1.docx]

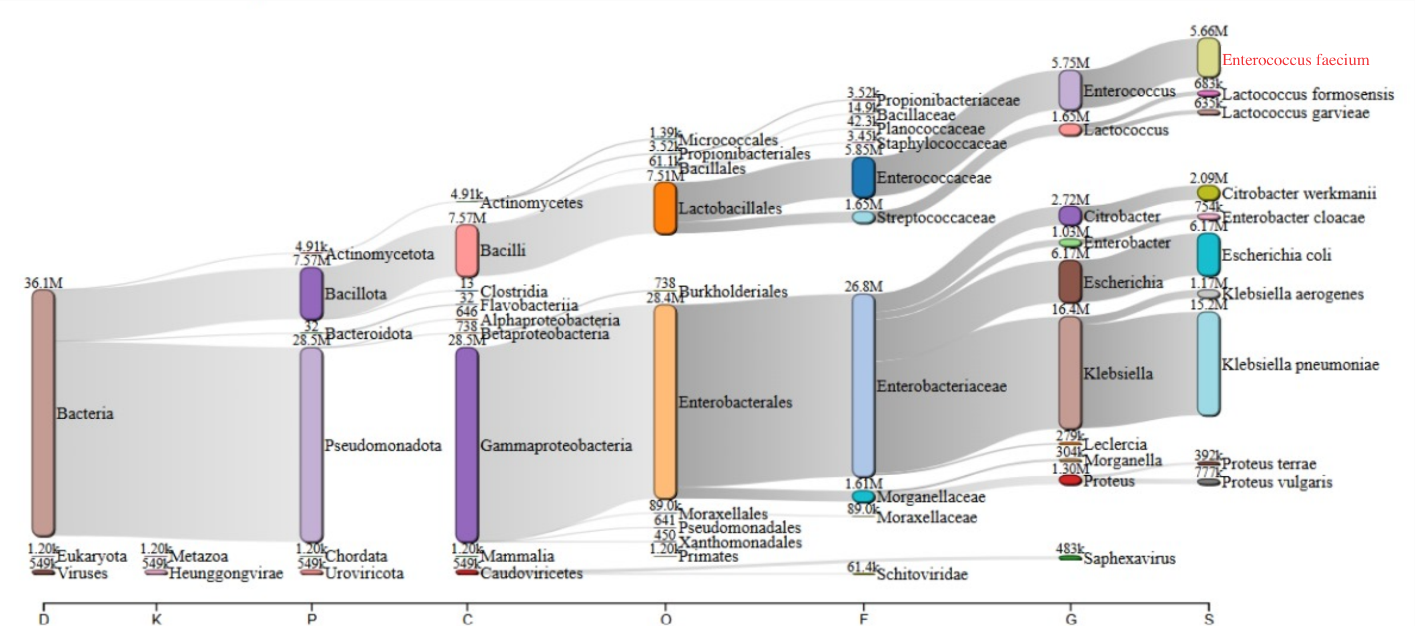


**Supplementary Data Figure S1:** Bins generated through binning analysis of whole genome sequenced data of current study documented bacterial mixture

**Supplementary Data Table S1:** Assembled genome analysis of *E. faecium* through Comprehensive Genome Analysis Service at PATRIC

| **Assembly details** | **Number** |
| --- | --- |
| Contigs | 129 |
| GC content | 38.00 |
| Plasmids | 0 |
| Contig L50 | 14 |
| Genome length | 2836039 bp |
| Contig N50 | 75082 |

**Supplementary Data Table S2:** Features of *E. faecium* genome annotated using RAST Tool kit

| **Annotated genome features** | **Number** |
| --- | --- |
| CDS | 2944 |
| tRNA | 33 |
| Partial CDS | 0 |
| rRNA | 0 |
| Miscellaneous RNA | 0 |
| Repeat regions | 0 |
| Job ID | annotation_804176 |
| Job started | July 4th 2025, 9:31:17am |
| Job completed | July 4th 2025, 9:36:51am |
| Total Time | 5 minutes and 34 seconds |

**Supplementary Data Table S3:** Hypothetical proteins and proteins with functional assignments identified in *E. faecium*

| **Protein features** | **Number** |
| --- | --- |
| Hypothetical proteins | 887 |
| Proteins with functional assignments | 2057 |
| Proteins with EC number assignments | 684 |
| Proteins with GO assignments | 560 |
| Proteins with pathway assignments | 462 |
| Proteins with PATRIC genus-specific family (PLfam) assignments | 2674 |
| Proteins with PATRIC cross-genus family (PGfam) assignments | 2813 |
